# Use of rebamipide solution as a submucosal injection material to prevent esophageal stricture after endoscopic submucosal dissection: Animal study

**DOI:** 10.1055/a-2820-3721

**Published:** 2026-04-14

**Authors:** Yuichiro Hirai, Ai Fujimoto, Motoki Sasaki, Masayuki Shimoda, Naohisa Yahagi

**Affiliations:** 1 38547 Department of Gastroenterology, National Hospital Organiszation Tokyo Medical Center, Tokyo, Japan; 2 Division of Research and Development for Minimally Invasive Treatment, Cancer Center, Keio University School of Medicine, Tokyo, Japan; 3 12839 Department of Pathology, The Jikei University School of Medicine, Tokyo, Japan

**Keywords:** Endoscopy Upper GI Tract, Endoscopic resection (ESD, EMRc, ...), Benign strictures, Ulcers (peptic and other)

## Abstract

**Background and study aims:**

It is desirable to develop a safe, cost-effective, and easy approach to prevent strictures after esophageal endoscopic submucosal dissection (ESD). We explored use of a novel rebamipide solution, an anti-ulcer drug, as a submucosal injection material for preventing esophageal stricture after ESD.

**Methods:**

In 15 swine, two half-circumferential ESDs were performed at the proximal and distal ends of the middle thoracic esophagus using 2% rebamipide solution or saline (control) as submucosal injection material. Five swine were sacrificed on postoperative days (PODs) 7, 14, and 21, respectively. Follow-up endoscopy was performed weekly until sacrifice. ESD-related outcomes, degree of stricture, and histological characteristics were evaluated.

**Results:**

ESD-related outcomes were similar in both groups, with all post-ESD ulcers epithelialized by POD 14. Mean mucosal constriction rate in the rebamipide and control groups on PODs 14 and 21 were 19.1 ± 7.2% vs. 21.0 ± 6.4% ( *P* = 0.40) and 13.3 ± 6.8% vs. 20.8 ± 6.1% ( *P* = 0.08), respectively. The rebamipide group peaked and showed milder constriction by POD 21, whereas the control group seemed to plateau. Histologically, mean fibrosis thickness in the rebamipide and control groups on days 14 and 21 were 807.0 ± 238.9 µm vs. 972.8 ± 395.1 µm ( *P* = 0.40) and 782.8 ± 281.5 µm vs. 1087.0 ± 476.1 µm ( *P* = 0.13), respectively.

**Conclusions:**

The rebamipide solution showed no statistically significant benefit in preventing post-ESD stricture. However, given the small sample size of this study, further studies are needed to clarify its potential role.

## Introduction

Endoscopic submucosal dissection (ESD), enabling en bloc resection with complete histological assessment, is standard treatment for superficial esophageal carcinoma 1 2 3 4 . However, esophageal strictures after extensive ESD remain a serious problem. Stricture occur in up to 80% to 100% of cases with more than three-quarters circumferential mucosal defects without preventive measures 5 6 . Strictures cause dysphagia and dietary restrictions, substantially burdening patients. Endoscopic balloon dilation (EBD) is commonly performed for strictures, but it often needs to be repeated long-term, which reduces quality of life and carries risks such as perforation 7 .

Several prophylactic approaches have been reported to alleviate strictures. Steroid therapy, including local injection and systemic administration, is the most widely used but cannot fully prevent strictures 4 8 . Furthermore, there are potential risks to local injection, such as tissue vulnerability and delayed perforation 9 , and severe infectious diseases have also been reported after systemic administration 10 .

Recently, efficacy of shielding mucosal defects with polyglycolic acid sheets and cell sheet transplantation has been explored 11 12 . However, there are barriers to use of these methods due to their complexity in preparation or delivery, and cell sheet transplantation can be particularly challenging owing to its inaccessibility and high cost 12 . Therefore, a safe, simple, and cost-effective approach to prevent post-ESD strictures is needed. In light of this, we focused on submucosal injection materials that we use during ESD and explored their potential for alleviating strictures.

Rebamipide (2-(4-chlorobenzoylamino)-3-[2-(1H)-quinolinon-4-yl]-propionic acid; Mucosta; Otsuka Pharmaceutical, Tokyo, Japan), is a widely prescribed agent for acute gastritis and gastric ulcers in Japan and other countries 13 . Our group previously showed that a 2% rebamipide solution used as submucosal injection material during gastric ESD promotes healing and may also suppress fibrosis in swine models 14 15 . We speculated that it may also be useful in preventing fibrosis after esophageal ESD. Hence, we investigated whether rebamipide solution as a submucosal injection material could safely prevent esophageal fibrosis and stricture after ESD in swine.

## Methods

### Study design

This was an experimental animal study using living swine. The protocol was approved by the Ethics Review Board of the Animal Experimental Laboratory of the Keio University, School of Medicine (number:17052). Fifteen domestic female swine weighing 20 to 25 kg were used. Esophageal ESDs were performed with a 2% rebamipide solution (rebamipide group) or a saline (control group) as submucosal injection material. ESD-related outcomes, stricture severity, and healing process were compared between the rebamipide and control groups based on endoscopic, macroscopic and histopathologic findings. In this study, rebamipide was diluted with 0.5% carboxymethyl cellulose (CMC; Wako Pure Chemical Industries, Ltd., Osaka, Japan). Rebamipide concentration was set at 2%, the maximum permissible level to maintain stability of the agent after dissolution. CMC served as a solvent for rebamipide because of its ability to maintain drug suspension and ensure consistent local delivery. Although CMC itself can influence wound healing and fibrosis 16 , our previous pilot experiments using a swine gastric ESD model (n = 2) suggested that rebamipide formulation suspended in CMC promoted ulcer healing and suppressed fibrosis more effectively than CMC alone, based on endoscopic and histopathologic evaluations. Therefore, normal saline was selected as the control because it is the standard submucosal injection material in clinical practice worldwide, highlighting the potential clinical relevance of the findings.

### Preoperative preparation

The swine were fasted 24 hours before ESD. ESD was performed under general anesthesia with intramuscular administration of midazolam (0.2 mg/kg), medetomidine (0.1 mg/kg), and atropine sulfate (0.02 mg/kg). Anesthesia was maintained with isoflurane inhalation, and an endotracheal tube was inserted to maintain respiration.

### ESD procedures

**Fig. 1:**
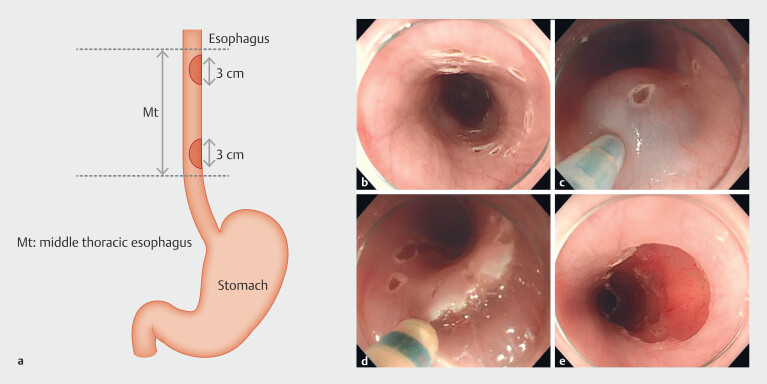
ESD procedure. **a** A 3-cm long, half-circumferential ESD is performed at 25 to 30 cm (proximal end) and 35 to 40 cm (distal end) from the incisors in each swine. **b** Marking dots are placed to outline the incision line. **c** As a submucosal injection material, 2% rebamipide solution is used at one site and saline solution at the other site, in a random order. All ESDs were performed by an experienced endoscopist blinded to the test solution. **d** A mucosal incision, just outside the marking dots and submucosal dissection are performed using a Dual knife J. **e** The artificial ulcer created by ESD is shown. ESD, endoscopic submucosal dissection

All procedures were performed in the left lateral decubitus position. A conventional endoscope (GIF-Q260J; Olympus, Tokyo, Japan) with a disposable distal attachment cap (D-201; Olympus) was used. For each swine, two artificial lesions with half-circumferential and 3-cm longitudinal diameters were created at 25 to 30 cm and 30 to 35 cm from the incisors ( Fig. 1 **a** ). To delineate margins of the hypothetical lesion, dots were marked using a 1.5-mm Dual knife J (KD-655Q; Olympus) and an electrosurgical unit (ESG-100; Olympus) ( Fig. 1 **b** ). ESD was performed using a 2% rebamipide solution at one site and saline at the other. The endoscopist was blinded to the solution used, whereas the assistant, who was not blinded, ensured alternate assignment of rebamipide and saline to the proximal and distal sites. All ESD procedures were conducted by a single experienced endoscopist (initials AF). Using a 23-gauge injection needle catheter (NM-600L-0423; Olympus), submucosal injection material was injected into the submucosa around the marking dots ( Fig. 1 **c** ), and a Dual knife J was used for mucosal incision and submucosal dissection ( Fig. 1 **d** , Fig. 1 **e** ). The pulse cut slow mode (30W) or forced coagulation mode (30W) was used for mucosal and submucosal incisions.

### Postoperative management and follow-up

**Fig. 2:**
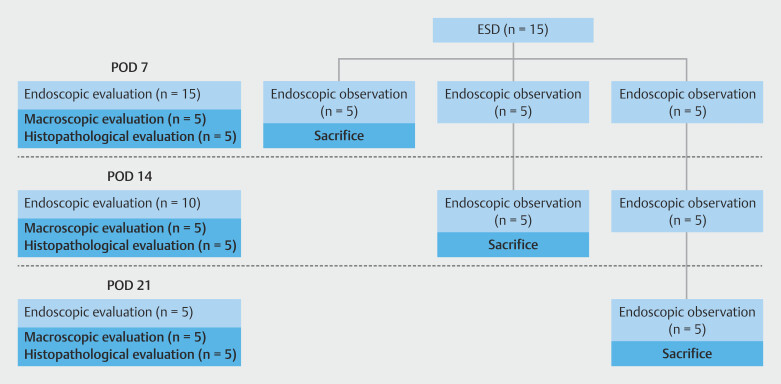
Study protocol.

The swine were fasted on the day of ESD and oral intake resumed on postoperative day (POD) 1. Postoperative conditions such as weight, daily food intake, and instances of vomiting were monitored. Swine with difficulty consuming the solid diet were fed a liquid or infused diet depending on their condition. Follow-up endoscopy was performed under general anesthesia using a conventional endoscope (9.9 mm in diameter, GIF-Q260J; Olympus) once a week until sacrifice. EBD or bougie was not performed even after stricture formation. Of the 15 swine, five each were sacrificed on PODs 7, 14, and 21, respectively. The swine were sacrificed by intravenous injection of 20-mL 15% potassium chloride (Terumo, Tokyo, Japan). After sacrifice, the esophagus was excised and opened longitudinally on the opposite side of the ESD scar and macroscopic and histopathologic evaluations were performed. The study protocol is shown in Fig. 2 .

### Histopathology and immunohistochemistry

The tissues were fixed in 10% neutral buffered formalin and the area of ESD ulcers was dissected along the short axis at 4-mm intervals. The slices were embedded in paraffin, cut into 4-μm-thick sections, and stained with hematoxylin and eosin and Azan-Mallory staining. Serial sections were cut for immunostaining using the mouse monoclonal anti-α-smooth muscle actin (α-SMA) antibody (1:400 dilution, 1A4/asm-1; Novus Biologicals, Littleton, Colorado, United States).

### Evaluation of outcomes

#### ESD-related outcomes

We evaluated the dose of the submucosal injection material, procedure time, en bloc resection rate, and adverse events (AEs), such as major bleeding and perforation, as ESD-related outcomes. Major bleeding was defined as massive bleeding requiring hemostatic forceps.

#### Endoscopic and macroscopic evaluations

During follow-up endoscopic examinations, wound condition and stricture development were assessed at each time point. To quantitatively analyze the degree of stricture, mucosal constriction rate was macroscopically evaluated using the excised esophagus after sacrifice. Mucosal constriction rate was calculated using previously reported formula 17 . [1- (length of the short axis of maximal constriction) / (length of the short axis at a normal mucosa on the proximal side + length of the short axis at a normal mucosa on the distal side)/2] ×100. Length of the short axis at the normal site was measured 15 mm away from the ulcer bed.

#### Histopathologic evaluation

To evaluate the ulcer healing process, thickness of the granulation tissue and the number of microvessels on POD 7 were assessed, along with widths of the absent muscularis mucosae on PODs 7, 14, and 21. Thickness of the granulation tissue was evaluated by Azan-Mallory staining using the following equation: area/width of the granulation tissue at the resection site, representing the mean vertical length of the granulation tissue. The numbers of microvessels were counted in three different and randomly selected fields (×400) of α-SMA sections and the values were averaged. Widths of absent muscularis mucosae were also measured in α-SMA sections.

To evaluate fibrosis formation, the proportion of α-SMA-positive cells, a marker for myofibroblasts, and thickness of fibrosis on PODs 7, 14, and 21 were assessed. The proportion of α-SMA-positive cells was quantified using Image J software (National Institutes of Health, Bethesda, Maryland, United States) in three different and randomly selected fields (×400) and the values were averaged. Thickness of fibrosis was measured by Azan- Mallory staining as: area /width of the fibrotic tissue at the resection site, representing the mean vertical length of the fibrotic tissue.

Furthermore, we evaluated damage to the muscularis propria in Azan-Mallory staining on a 4-point scale, as previously reported 16 : 0 = no atrophic or fibrotic change; 1 = atrophic or fibrotic change confined to the internal circular muscularis propria; 2 = atrophic or fibrotic change confined to the external longitudinal muscularis propria; and 3 = transmural fibrosis.

A representative section from the central part of the wound was evaluated using a virtual pathology system (Nanozoomer Digital Pathology, Hamamatsu Photonics, Hamamatsu, Japan) for all outcomes.

### Statistical analysis

Because this study was the first exploratory study to investigate efficacy and safety of the rebamipide solution as submucosal injection material for preventing stricture after esophageal ESD, no specific assumptions regarding sample size were made. We chose to use a total of 15 swine to evaluate histopathological findings of five swine at each time point (PODs 7, 14, and 21). This was a pragmatic choice based on our experience with similar gastric ESD experiments in swine 14 15 . Data are presented as percentages for categorical variables and mean ± standard deviation (SD) for continuous variables. Categorical variables were compared between groups using the chi-square test or Fisher exact test, as appropriate. Continuous variables were compared between groups at individual time points using the Wilcoxon rank-sum test. In addition, linear mixed-effects models with group, time, and their interaction as fixed effects and subject as a random intercept were used to evaluate changes in outcomes at PODs 14 and 21 relative to POD 7 between groups. *P* < 0.05 was considered statistically significant and all statistical analyses were performed using JMP version 16.0 (SAS Institute Inc, Cary, North Carolina, United States).

## Results

### ESD-related outcomes

ESD-related outcomes are presented in Table 1 . All ESDs were successfully performed en bloc in both groups. Mean doses of submucosal injection material were 30.8 ± 9.1 mL and 27.5 ± 7.3 mL in the rebamipide and control groups, respectively ( *P* = 0.45), and mean procedure times were 20.0 ± 5.7 minutes and 18.7 ± 5.0 minutes, respectively ( *P* = 0.55). No massive bleeding or perforation occurred and vital signs remained stable throughout the procedures in either group.

**Table:** **Table 1** Details of ESD-related outcomes.

	**Rebamipide (n = 15)**	**Control (n = 15)**	***P* value **
Does of submucosal injection material, mean (SD), mL	30.8 (9.1)	27.5 (7.3)	0.45
Procedure time, mean (SD), min	20.0 (5.7)	18.7 (5.0)	0.55
En bloc resection, n (%)	15 (100)	15 (100)	
Major bleeding, n (%)	0 (0)	0 (0)	
Perforation, n (%)	0 (0)	0 (0)	
Other adverse events, n (%)	0 (0)	0 (0)	
ESD, endoscopic submucosal dissection; SD, standard deviation			

### Endoscopic and macroscopic evaluations

**Fig. 3:**
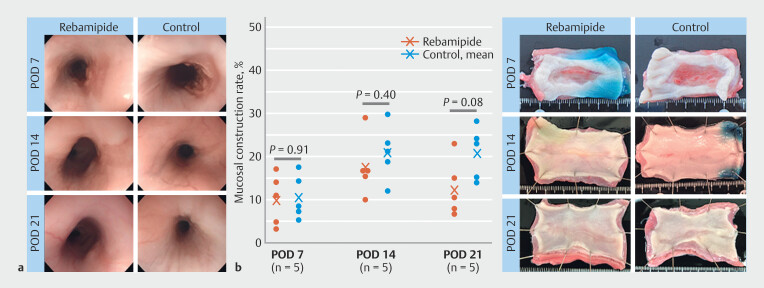
Endoscopic and macroscopic evaluations. **a** Representative images of the follow-up endoscopic examination in the rebamipide and control groups on PODs 7, 14 and 21. The wounds remained open on POD 7 and were fully epithelialized on POD 14 in all swine of both groups. By POD 14, mild strictures had developed in both groups although they appeared less severe in the rebamipide group compared to the control group. **b** Mucosal constriction rate and representative images of the extracted esophagus in the rebamipide and control groups on PODs 7, 14, and 21. No significant differences in mucosal constriction rates were observed between the rebamipide and control groups on POD 7 (10.0 ± 5.9% vs. 10.5 ± 5.2%, P = 0.91), POD 14 (19.1 ± 7.2% vs. 21.0 ± 6.4%, P = 0.40), or POD 21 (13.3 ± 6.8% vs. 20.8 ± 6.1%, P = 0.08). Linear mixed‑effects analysis likewise detected no significant group–time interaction. However, the constriction rate peaked and subsequently declined by POD 21 in the rebamipide group, whereas it plateaued in the control group. POD, postoperative day

Endoscopic evaluation revealed that wounds were still open on POD 7 and completely epithelialized on POD 14 in all swine in both groups. By POD 14, mild strictures had developed in both groups, but appeared less severe in the rebamipide group compared with the control group ( Fig. 3 **a** ). Macroscopic evaluation showed no significant differences in mucosal constriction rates between the rebamipide and control groups on POD7 (10.0 ± 5.9% vs. 10.5 ± 5.2%, respectively. *P* = 0.91), POD14 (19.1 ± 7.2% vs. 21.0 ± 6.4%, respectively, *P* = 0.40), and POD 21(13.3 ± 6.8% vs. 20.8 ± 6.1%, respectively, *P* = 0.08) ( Fig. 3 **b** ). In addition, linear mixed-effects analysis showed no significant group-time interaction. However, the mucosal constriction rate in the rebamipide group peaked and became milder on POD 21, whereas it plateaued in the control group.

### Histopathologic evaluation

**Fig. 4:**
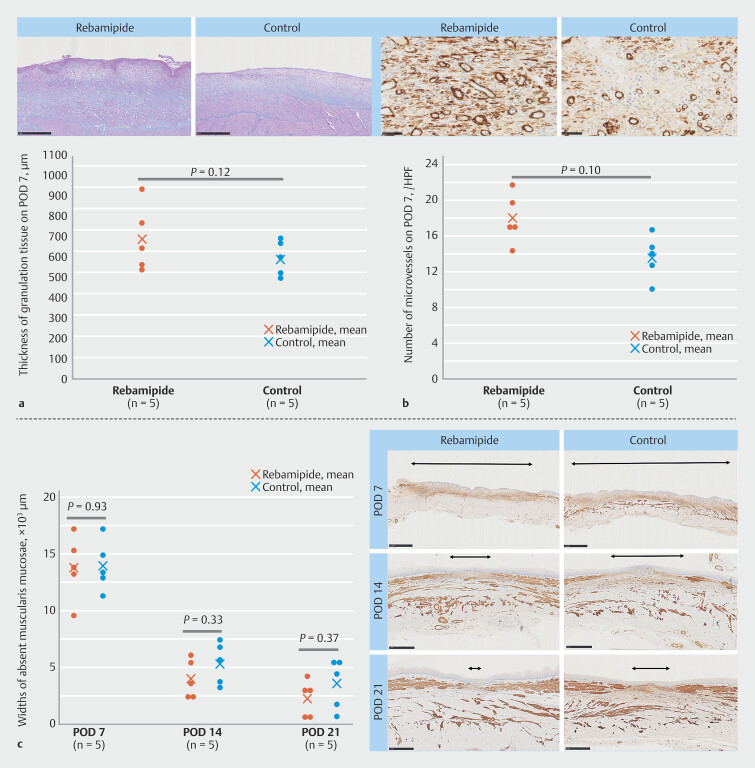
Histopathologic evaluation of ulcer healing. **a** Thickness of granulation tissue and representative images of Azan-Mallory staining in the rebamipide and control groups on POD 7 (Scale bar, 1000 µm). The rebamipide group exhibited a greater thickness of granulation tissue compared with the control group (718.0 ± 144.0 µm vs. 589.6 ± 84.9 µm), although this difference did not reach statistical significance (P = 0.12). **b** The number of microvessels and representative images of α-SMA sections in the rebamipide and control groups on POD 7 (Scale bar, 50 µm). Although the rebamipide group showed a greater number of microvessels (17.0 ± 3.4 vs. 13.8 ± 1.5), the difference was not statistically significant (P = 0.10). **c** Widths of absent muscularis mucosae and representative images of Azan Mallory staining in the rebamipide and control groups on PODs 7, 14 and 21 (Scale bar, 2500 µm for POD 7; 1000 µm for PODs 14 and 21). Mean widths in the rebamipide and control groups on PODs 7, 14, and 21 were 13.8 ± 2.8 ×10³/µm vs. 13.9 ± 2.2 ×10³/µm (P = 0.93), 4.0 ± 1.7 ×10³/µm vs. 5.3 ± 1.9 ×10³/µm (P = 0.33), and 2.3 ± 1.6 ×10³/µm vs. 3.5 ± 2.2 ×10³/µm (P = 0.37), respectively. Although widths tended to be shorter in the rebamipide group on PODs 14 and 21, no statistically significant differences were detected. Linear mixed‑effects analysis revealed no significant group-time interaction. α-SMA, α-smooth muscle actin; POD, postoperative day.

Histologically, epithelialization had already begun on POD 7 and was completely epithelialized on POD 14 in all swine in both groups. On POD 7, mean thicknesses of granulation tissue were 718.0 ± 144.0 µm and 589.6 ± 84.9 µm in the rebamipide and control groups, respectively ( *P* = 0.12) ( Fig. 4 **a** ). Mean numbers of microvessels were 17.0 ± 3.4 and 13.8 ± 1.5 in the rebamipide and control groups, respectively ( *P* = 0.10) ( Fig. 4 **b** ). On POD 14, granulation tissue and microvessels were no longer prominent after epithelialization was completed. Mean widths of absent muscularis mucosae in the rebamipide and control groups on PODs 7, 14, and 21 were 13.8 ± 2.8 ×103/µm vs. 13.9 ± 2.2 ×103/µm (P = 0.93); 4.0 ± 1.7 ×103/µm vs. 5.3 ± 1.9 ×103/µm (P = 0.33); and 2.3 ± 1.6 ×103/µm, vs. 3.5 ± 2.2 ×103/µm (P = 0.37), respectively ( Fig. 4 **c** ). Although not statistically significant, thickness of the granulation tissue and number of microvessels were greater on POD 7 and widths of absent muscularis mucosae were shorter on PODs 14 and 21 in the rebamipide group than in the control group. Linear mixed-effects analysis also showed no significant group-time interaction.

**Fig. 5:**
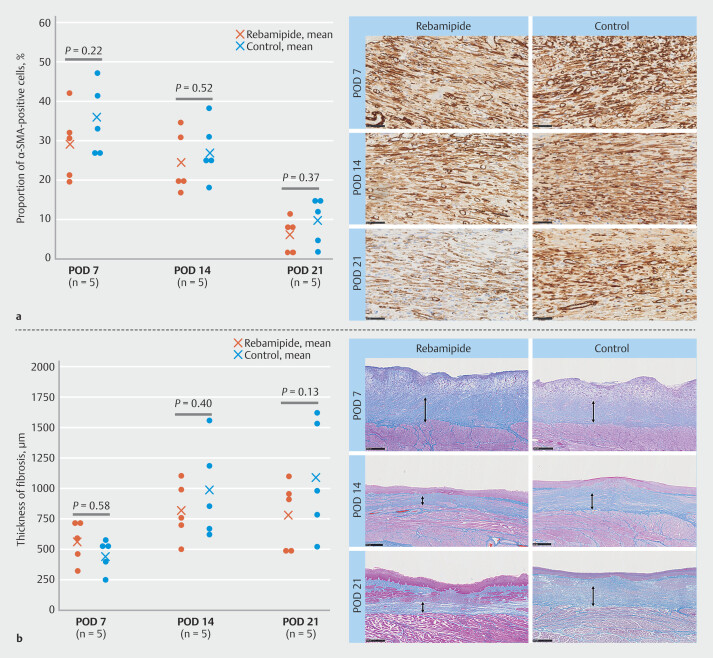
Histopathologic evaluation of fibrosis formation. **a** Proportion of α-SMA-positive cells and representative images of α-SMA sections in the rebamipide and control groups on PODs 7, 14, and 21 (Scale bar, 50 µm). Proportions of α‑SMA–positive cells in the rebamipide and control groups on PODs 7, 14, and 21 were 29.0 ± 9.1% vs. 35.1 ± 9.0% (P = 0.22), 24.3 ± 7.9% vs. 27.4 ± 7.5% (P = 0.52), and 19.2 ± 2.2% vs. 25.8 ± 7.4% (P = 0.18), respectively ( **a** ). Although none of these differences were statistically significant, the rebamipide group consistently showed lower proportions of α‑SMA–positive cells across all time points. Linear mixed‑effects analysis revealed no significant group–time interaction. **b** Thickness of fibrosis and representative images of Azan-Mallory staining in the rebamipide and control groups on PODs 7, 14, and 21 (Scale bar, 500 µm). Thickness of fibrosis in the rebamipide and control groups on PODs 7, 14, and 21 was 558.6 ± 169.7 µm vs. 450.8 ± 131.1 µm (P = 0.58), 807.0 ± 238.9 µm vs. 972.8 ± 395.1 µm (P = 0.40), and 782.8 ± 281.5 µm vs. 1087.0 ± 476.0 µm (P = 0.13), respectively. Fibrosis progressed on POD 7 but was attenuated on PODs 14 and 21 in the rebamipide group compared with the control group. Linear mixed‑effects analysis demonstrated a significant group–time interaction, with the between‑group difference becoming evident at POD 21 (P = 0.049). α-SMA, α-smooth muscle actin; POD, postoperative day

Proportions of α-SMA-positive cells in the rebamipide and control groups on PODs 7, 14, and 21 were 29.0 ± 9.1% vs. 35.1 ± 9.0% ( *P* = 0.22); 24.3 ± 7.9% vs. 27.4 ± 7.5% ( *P* = 0.52); and 19.2 ± 2.2% vs. 25.8 ± 7.4% ( *P* = 0.18), respectively ( Fig. 5 **a** ). Thickness of fibrosis in the rebamipide and control groups on PODs 7, 14, and 21 were 558.6 ± 169.7 µm vs. 450.8 ± 131.1 µm ( *P* = 0.58); 807.0 ± 238.9 µm vs. 972.8 ± 395.1 µm ( *P* = 0.40); and 782.8 ± 281.5 µm vs. 1087.0 ± 476.0 µm ( *P* = 0.13), respectively ( Fig. 5 **b** ). None of these findings were statistically significant, but the rebamipide group had a smaller proportion of α-SMA-positive cells than the control group at all time points. Fibrosis was progressive on POD 7 but was attenuated on PODs 14 and 21 in the rebamipide group compared with the control group. Linear mixed-effects analysis showed no significant group-time interaction for the proportion of α-SMA-positive cells, whereas a significant group-time interaction was observed for fibrosis thickness, with the between-group difference becoming apparent at POD 21 ( *P* = 0.049).

Mean scores for damage to the muscularis propria in the rebamipide and control groups on PODs 7, 14, and 21 were 1.2 ± 0.4 vs. 1.4 ± 0.8 ( *P* = 0.67); 1.4 ± 1.1 vs. 2.0 ± 0.8 ( *P* = 0.40); and 1.2 ± 0.4 vs. 1.4 ± 0.5 ( *P* =0.55), respectively ( **Supplementary Fig. 1** ).

## Discussion

This in vivo study investigated whether a novel rebamipide solution as a submucosal injection material could safely promote ulcer healing to prevent strictures after esophageal ESD. ESD-related outcomes were comparable between groups, whereas endoscopic and macroscopic findings suggested milder strictures in the rebamipide group. Histopathological findings suggested accelerated ulcer healing and less severe fibrosis in the rebamipide group. However, no statistically significant reduction in stricture was observed by POD 21, possibly due to the small sample size. Nevertheless, the rebamipide group exhibited a 7.5% lower mucosal constriction rate and consistent trends toward reduced fibrosis. These findings require confirmation in larger studies.

Mechanisms of ulcer healing leading to stricture formation are not fully understood; however, reduced elasticity due to inflammation and fibrosis is considered to cause post-ESD stricture 16 17 . We hypothesized that the potential means to prevent stricture is to ameliorate inflammation, leading to early induction of the proliferation and remodeling phases in the wound healing process. Although rebamipide is commercially available in Japan for gastritis and as an eye drop for dry eyes 18 , its anti-ulcer action has been explored for various purposes 19 20 21 , including use as a gargle for chemoradiotherapy-induced oral mucositis 21 . In animal models, rebamipide has been shown to promote tissue repair in various organs 22 23 24 . It acts by decreasing oxygen radicals, increasing blood flow, and producing endogenous prostaglandins (PGs) 25 , which accelerate healing. Rebamipide also aids tissue replacement by upregulating epidermal growth factor and its receptors, promoting ulcer healing through angiogenesis and increasing granulation tissue formation 26 27 . In our study, the rebamipide group showed thicker granulation tissue and increased microvessels on POD 7 compared with the control group, as well as shorter width of the absent muscularis mucosae on PODs 7, 14, and 21, indicating accelerated ulcer healing.

Meanwhile, fibroblast-to-myofibroblast transformation is widely accepted as a key event in tissue repair 28 29 . The high contractile force of myofibroblasts aids wound closure; however, prolonged or excessive activity leads to fibrosis 28 29 . A rat study suggested that increased PGE2 levels induced by rebamipide suppress hepatic fibrosis 30 because PGE2 is considered a potent suppressor of fibrosis that attenuates fibroblast-to-myofibroblast differentiation and limits myofibroblasts migration 31 32 . Here, rebamipide appeared to reduce excessive myofibroblasts as indicated by decreased α-SMA-positive cells, and this may have led to suppression of fibrosis. At POD 7, although the proportion of α-SMA-positive cells was lower in the rebamipide group, fibrosis was more pronounced. This discrepancy may reflect differences in timing of the wound healing process. Generally, α-SMA transiently increases during the active wound contraction phase and decreases as healing progresses. Therefore, accelerated healing in the rebamipide group may have caused the peak of α-SMA expression to occur before POD7, resulting in a decline at observation, whereas fibrosis formation had already started. A linear mixed-effects analysis across the observation period suggested a group-by-time interaction in fibrosis thickness, indicating different temporal patterns of fibrotic remodeling between groups. Consistent with these findings, after epithelialization from POD 14 onward, fibrosis tended to increase in the control group, but remained similar in the rebamipide group. Importantly, despite similar fibrosis levels in the rebamipide group between POD 14 and POD 21, mucosal constriction decreased by POD 21. This suggests that suppression of excessive myofibroblast contraction by rebamipide also contributed to preserving elasticity of the healing tissue, potentially facilitating easier-to-perform EBD even if stricture develops.

Although the precise mechanisms by which steroids, the most common therapy, prevent stricture are not fully elucidated, they are thought to act through anti-inflammatory effects and enhanced collagenase activity. However, their potent actions are associated with delayed ulcer healing and tissue fragility when locally injected, and may also cause excessive damage to the muscularis propria through catabolic effects. By contrast, rebamipide appeared to provide faster ulcer healing and caused no notable injury to the muscularis propria when used as a submucosal injection material. Although safety data on rebamipide injection are currently limited to our study, the oral and ophthalmic formulations are widely used, and its mechanism of action supports a favorable safety profile. Investigating whether combining steroids with rebamipide yields synergistic benefits or counteracts their actions may also be valuable.

In recent decades, various approaches have been attempted to prevent post-ESD esophageal strictures, but most are applied to ulcers subsequently after ESD 8 11 12 . These approaches increase procedure time and complexity, and may increase risk of AEs. Submucosal injection during ESD is routinely used to lift lesions for safe dissection. Although fluids such as glycerin/fructose and sodium hyaluronate have been investigated to maintain submucosal elevation 33 , few studies have examined submucosal injection materials with simultaneous tissue repair functions. For example, electric current during submucosal dissection can cause inflammation and several studies suggested that the degree of fibrosis and stricture may differ depending on the electrosurgical mode 34 35 . Using an anti-ulcer drug such as our rebamipide solution as a submucosal injection material may reduce thermal injury and inflammation, although duration and distribution of the active ingredients within the limited area of the esophageal submucosa during healing remain unclear. However, the idea of using submucosal injection material as a measure to alleviate post-ESD stricture is acceptable because it requires no extra effort. Considering that rebamipide is inexpensive, this solution merits further evaluation. Future experimental studies using various solvents to enhance solubility or stability may expand the potential of rebamipide.

This study has several limitations. First, we used a semi-circular ESD model. Although more aggressive models, such as full circumferential ESD, better represent severe strictures, they often cause pinhole stenosis, impaired food intake, and even death. Although our model produced only mild stricture formation, even in the control group, it still allowed us to capture trends in histopathological changes and stricture progression across groups. Second, we created two lesions in a single swine, and mutual influence cannot be excluded. In addition, stricture-related symptoms, including weight loss, food intake, and dysphagia, could not be compared between groups. Ideally, one lesion per swine should be prepared; however, this would require many swine and raise ethical concerns. Third, the potential influence of CMC, used as a solvent for rebamipide, on the ulcer healing process remains uncertain. Comparing with CMC alone would have been useful to clarify its specific contribution. Nevertheless, our study prioritized comparison with the submucosal injection material commonly used in clinical practice, where normal saline is the standard. Although ESD outcomes were similar between the groups, the higher viscosity of CMC could theoretically favor the procedure, which should be considered when interpreting the results. Lastly, our study lacked long-term outcomes (> POD 21). Although previous swine studies reported that esophageal post-ESD strictures develop between PODs 14 and 21 16 17 , time course changes after stricture formation require further evaluation.

## Conclusions

In conclusion, this study did not demonstrate a statistically significant effect of rebamipide solution as a submucosal injection material on ulcer healing or fibrosis, possibly due to the small sample size. Nevertheless, consistent temporal patterns were observed across endoscopic, macroscopic, and histological evaluations. Although these findings do not allow definitive conclusions, they provide insights for future studies evaluating the impact of different submucosal injection materials on ulcer healing and fibrosis. This study represents a preliminary preclinical investigation guiding further research under optimized conditions.

## Supplementary Material

Supplementary Material Zusatzmaterial
