# Supplementary material for: Use of rebamipide solution as a submucosal injection material to prevent esophageal stricture after endoscopic submucosal dissection: Animal study
Source: Endosc Int Open. 2026 Apr 14;14:a28203721. doi: 10.1055/a-2820-3721 (PMC13093119; doi:10.1055/a-2820-3721)
Supplement: Supplementary file 1 — Supplementary Material [file 10-1055-a-2820-3721_28337552.pdf]

Supplementary Fig. 1 Muscularis propria damage score in the rebamipide and control groups on PODs 7, 14, and 21. Mean scores in the rebamipide and control groups on PODs 7, 14, and 21 were 1.2 ± 0.4 vs. 1.4 ± 0.8 (P = 0.67); 1.4 ± 1.1 vs. 2.0 ± 0.8 (P = 0.40); and 1.2 ± 0.4 vs. 1.4 ± 0.5 (P = 0.55), respectively.

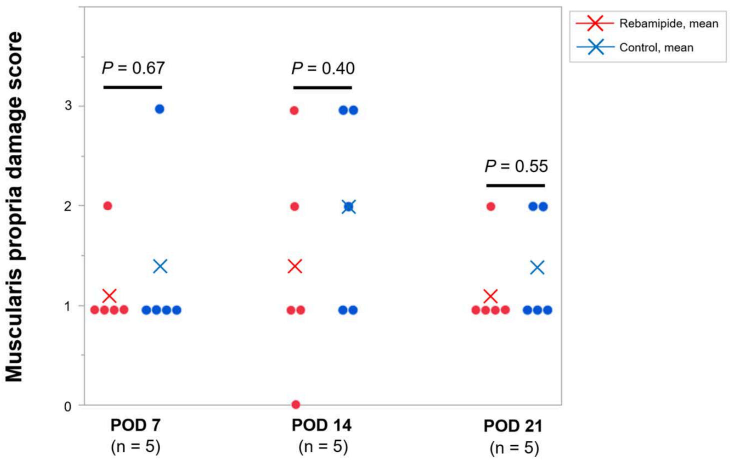

Endosc Int Open | DOI 10.1055/a-2820-3721 | © The Author(s).
